# Biological synthesis of ursodeoxycholic acid

**DOI:** 10.3389/fmicb.2023.1140662

**Published:** 2023-02-24

**Authors:** Peng Song, Xue Zhang, Wei Feng, Wei Xu, Chaoyun Wu, Shaoqing Xie, Sisi Yu, Rongzhao Fu

**Affiliations:** ^1^College of Life Sciences, Liaocheng University, Liaocheng, China; ^2^Jiangxi Zymerck Biotechnology Co., Ltd., Nanchang, China

**Keywords:** ursodeoxycholic acid, chenodeoxycholic acid, biological synthesis, free-enzyme catalysis, whole-cell synthesis

## Abstract

Ursodeoxycholic acid (UDCA) is a fundamental treatment drug for numerous hepatobiliary diseases that also has adjuvant therapeutic effects on certain cancers and neurological diseases. Chemical UDCA synthesis is environmentally unfriendly with low yields. Biological UDCA synthesis by free-enzyme catalysis or whole-cell synthesis using inexpensive and readily available chenodeoxycholic acid (CDCA), cholic acid (CA), or lithocholic acid (LCA) as substrates is being developed. The free enzyme-catalyzed one-pot, one-step/two-step method uses hydroxysteroid dehydrogenase (HSDH); whole-cell synthesis, mainly uses engineered bacteria (mainly *Escherichia coli*) expressing the relevant HSDHs. To further develop these methods, HSDHs with specific coenzyme dependence, high enzyme activity, good stability, and high substrate loading concentration, P450 monooxygenase with C-7 hydroxylation activity and engineered strain harboring HSDHs must be exploited.

## Introduction

Ursodeoxycholic acid (UDCA) is an endogenous bile acid that has been shown to dissolve gallstone (Park et al., 2020; Haal et al., 2021); treat cholestasis (Leoni et al., 2018; Marschall, 2019; Razori et al., 2019; Chappell et al., 2020; Linhares et al., 2020; Haal et al., 2021; Kato et al., 2022), biliary pancreatitis (Rose et al., 1991; Testoni et al., 2000; Byelyayeva, 2015), primary cholangitis (Carbone et al., 2018; Deneau et al., 2019; Cacciato et al., 2020; Qian et al., 2020; Yao et al., 2020; Yu et al., 2021), colitis (colon cancer) (Eun-Kyung et al., 2017; He et al., 2022), (non)alcoholic hepatitis (Khachidze, 2019; Wu et al., 2020; Xie et al., 2022), primary biliary cirrhosis (van der Feen et al., 2016; Ye et al., 2019; Colombo et al., 2021), and drug-induced hepatitis (Karim and Yesmin, 2020); and improve liver transplantation outcomes (Harms et al., 2019; Kato et al., 2022), while gut microbiota-derived UDCA also attenuates colon inflammation in low-birth-weight infants by enhancing macrophage M2 polarization (Pi et al., 2022). In recent years, it has been found that UDCA can also mediate and influence the regulation of oncogenic signaling pathways in the cancer genome for cancer therapy (Goossens and Bailly, 2019; Lugini et al., 2022); it can specifically regulate the threshold of apoptosis, inhibit cancer cell growth (Eun-Kyung et al., 2017; Chen et al., 2022), and induce autophagy and apoptosis (Lim and Han, 2015; Lee et al., 2017). It has also been found that UDCA can improve damaged mitochondrial function and exert neuroprotective effects in the treatment of progressive neurological diseases (Bell et al., 2018; Abdelkader, 2020; Qi et al., 2020; Sathe et al., 2020; West et al., 2020; Yang et al., 2020; Huang, 2021).

Originally discovered as a natural active product from polar bear bile and named by Hammarsten in 1902, UDCA has gradually been shown to exhibit better efficacy in the treatment of gallbladder and liver-related diseases than other endogenous bile acids (Beuers et al., 1992; Combes et al., 1995; Im and Martinez, 2004). To date, UDCA is the only drug approved by the U.S. Food and Drug Administration (FDA) for the treatment of primary biliary cirrhosis (Shah and Kowdley, 2020). UDCA is now prepared by chemical or biosynthetic routes: the chemical route produces UDCA by a seven-step synthesis using cholic acid (CA) or chenodeoxycholic acid (CDCA) as starting substrates (Hofmann et al., 1963), with CA and CDCA isolated from inexpensive and readily available bovine bile and chicken, duck, and goose bile, respectively. Unfortunately, this route requires the use of toxic and dangerous hydrazine, CrO_3_ and pyridine reagents and generates a large amount of waste, in addition to an overall yield of only approximately 30%. To improve the efficiency of UDCA synthesis, alternative routes were developed, and Dangate et al. (2011) used o-iodobenzoic acid, hydrazine and sodium metal dissolved in n-propanol in the reaction to circumvent the group protection and deprotection steps of the original process and increase the yield of CA to UDCA to 53%; however, this preparation process is still not optimized in terms of cost and environmental protection. UDCA produced by chemical synthesis is still far from meeting market demand in terms of both quantity and quality (Tonin and Arends, 2018). Compared with chemical synthesis, biosynthesis is greener, efficient and safe, and UDCA biosynthesis using bile acid substrates is gradually being developed.

Biological UDCA synthesis is mainly free enzyme-catalyzed synthesis or whole-cell synthesis (Eggert et al., 2014). Free enzyme-catalyzed synthesis uses CDCA or CA as a substrate, and UDCA is synthesized through a multienzyme cascade reaction; whole-cell synthesis involves the addition of CDCA or lithocholic acid (LCA) substrate during microbial culture, which is converted into UDCA by microbial cells.

## UDCA synthesis catalyzed by free enzymes

Compared with whole-cell UDCA synthesis, free enzyme-catalyzed UDCA synthesis has been more extensively studied. Free enzyme-catalyzed UDCA synthesis uses CDCA, CA or LCA as starting substrates, with most of the studies focusing on CDCA and CA substrates. On the one hand, CDCA and CA are isolated from poultry (chicken, duck, and goose) and bovine bile, and the raw materials are inexpensive and easily available; on the other hand, the catalytic efficiency of hydroxysteroid dehydrogenase (HSDH) for the conversion of CDCA and CA into UDCA is relatively high. Currently, only CDCA substrate is used for free enzyme-catalyzed UDCA synthesis, and UDCA synthesis can also be catalyzed by substrate-specific HSDH using CA as substrate, but the key step of this synthetic route still requires a chemical reaction, i.e., the reduction of the intermediate product carbonyl (C=O) to methylene using the Wolff–Kishner reaction (CH_2_). Using LCA as a substrate, the conversion to UDCA requires a C-7 carbonyl oxygenation reaction *via* P450 monooxygenase.

### CDCA as a substrate for UDCA synthesis

**FIGURE 1:**
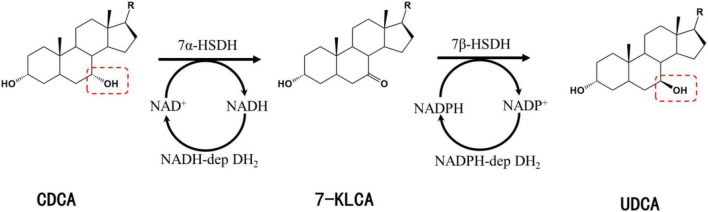
Synthesis of UDCA from CDCA catalyzed by 7α-HSDH and 7β-HSDH. R, 4-pentynoic acid; red dashed box, C-7 hydroxyl group.

Chenodeoxycholic acid is converted into UDCA upon the isomerization of the C-7 hydroxyl group. UDCA synthesis using CDCA as a substrate involves two key enzymes: 7α-hydroxysteroid dehydrogenase (7α-HSDH) and 7β-hydroxysteroid dehydrogenase (7β-HSDH). 7α-HSDH first catalyzes the oxidative dehydrogenation of CDCA to produce 7-ketolithocholic acid (7-KLCA), and 7β-HSDH then catalyzes the hydrogenation and reduction of 7-KLCA to produce UDCA (Figure 1). 7α-HSDH specifically oxidizes the α-hydroxyl group at C7 and reduces NAD^+^/NADP^+^, while 7β-HSDH specifically reduces the hydroxyl group at C7 to generate the β-hydroxyl group and oxidizes NADPH or NADH (Figure 1).

7α-Hydroxysteroid dehydrogenase and 7β-HSDH belong to the same short-chain dehydrogenase/reductase (SDR) family with a molecular mass of approximately 30 kDa and generally exist in homodimeric or homotetrameric structures (Oppermann et al., 2003; Kavanagh et al., 2008). The first 7α-HSDHs were isolated from aerobic or anaerobic bacteria, and in recent years, a larger number of 7α-HSDHs have been identified from black bear feces by homologous alignment (Ji et al., 2014, 2021; Tang et al., 2019) or metagenomics methods (Song et al., 2017). The cofactor-dependent and enzymatic activities of the reported 7α-HSDHs are shown in Table 1.

**TABLE 1:** The reported 7α-HSDHs.

Number	Sequence ID	Microbial source	Cofactor	Enzyme activity (U/mg)	References
1	GenBank: WP_046653274.1	*Brevundimonas* sp.	NAD^+^	471.0	Chen et al., 2019
2	GenBank: MH743112	Uncultured bacterium (from fecal samples of black bear)	NADP^+^	20	Tang et al., 2019
3	GenBank: MG516982	Uncultured bacterium (from fecal samples of black bear)	NADP^+^	188.3	Ji et al., 2021
4	GenBank: KF356399	*Comamonas testosteroni*	NADP^+^	∼185	Ji et al., 2014
5	S1-a-1	Fecal samples of black bears	NADP^+^	/	Song et al., 2017
6	S1-a-2	Fecal samples of black bears	NADP^+^	/	Song et al., 2017
7	H1-a-1	Fecal samples of black bears	NADP^+^	/	Song et al., 2017
8	H1-a-2	Fecal samples of black bears	NADP^+^	/	Song et al., 2017
9	Y1-a-1	Fecal samples of black bears	NADP^+^	/	Song et al., 2017
10	GenBank: AAA53556.1	*Clostridium sordellii*	NADP^+^	1.1	Coleman et al., 1994
11	GenBank: AAB61151.1	*Eubacterium* sp.	NADP^+^	338	Baron et al., 1991
12	GenBank: JN191345.1	*Clostridium absonum*	NADP^+^	59	Elisa et al., 2012
13	GenBank: WP_011860631.1	*Clostridium difficile*	NADP^+^	8.5	Bakonyi and Hummel, 2016
14	GenBank: KXH01569.1	*Escherichia coli*	NAD^+^	190	Yoshimoto et al., 1991
15	GenBank: OGX95366.1	*Bacteroides fragilis*	NAD^+^	351	Bennett et al., 2003
16	/	*Xanthomonas maltophilia*	NAD^+^	70	Pedrini et al., 2005

In addition to the above functionally validated 7α-HSDHs, a search for “7α-hydroxysteroid dehydrogenase” in the NCBI RefSeq database yielded more than 1,200 entries (O’Leary et al., 2016). In addition, some processes related to 7α-HSDH mutants or selective oxidation, derivative synthesis, and salt formation reactions have been patented (Gupta et al., 2009; Monti et al., 2012; Schmid et al., 2013; Bakonyi et al., 2020).

Unlike 7α-HSDH, 7β-HSDH has not been used as frequently for biotransformation (Table 2). Moreover, the specific enzymatic activity and enzymatic stability of 7β-HSDH are poor (Hummel and Bakonyi, 2016; Zheng et al., 2017b), and only one wild-type 7β-HSDH derived from *Clostridium absonum* was used in two different UDCA synthesis processes (Bovara et al., 1996; Monti et al., 2011). 7β-HSDH almost always requires NADPH as a coenzyme, and thus far, only two NAD^+^-dependent 7β-HSDHs have been identified: one from *Lactobacillus spicheri* (Tonin et al., 2018) and one from *Xanthomonas maltophilia* with a specific enzymatic activity of 33 U/mg, but the protein sequences have not been reported (Pedrini et al., 2005). To improve the activity and stability of 7β-HSDHs, numerous studies have focused on the protein engineering of 7β-HSDH to obtain mutants that meet industrial application requirements (Hummel and Bakonyi, 2016; Liu et al., 2016, 2018, 2019; Zheng et al., 2017b).

**TABLE 2:** The reported 7β-HSDHs.

Number	Sequence ID	Microbial source	Cofactor	Enzyme activity (U/mg)	References
1	GenBank: JN191345.1	*Clostridium absonum*	NADPH	65	Elisa et al., 2012
2	/	*Xanthomonas maltophilia*	NADH	33	Pedrini et al., 2005
3	GenBank: ZP0177306.1	*Collinsella aerofaciens*	NADPH	30	Liu et al., 2011
4	GenBank: ZP02041813	*Ruminococcus gnavus*	NADPH	23	Lee et al., 2013
5	GenBank: WP015528793	*Ruminococcus torques*	NADPH	8.6	Zheng et al., 2017b
6	Engineered	*Ruminococcus torques*	NADPH	46.8	Zheng et al., 2017b
7	GenBank: WP006236005	*Collinsella aerofaciens*	NADPH	15	Hummel and Bakonyi, 2016
8	Engineered	*Collinsella aerofaciens*	NADPH	21	Hummel and Bakonyi, 2016
9	Y1-b-1	Fecal samples of black bears	NADPH	/	Song et al., 2017
10	WP_045806907.1	*Lactobacillus spicheri*	NADH	3.1	Tonin et al., 2018

### CA as a substrate for UDCA synthesis

**FIGURE 2:**
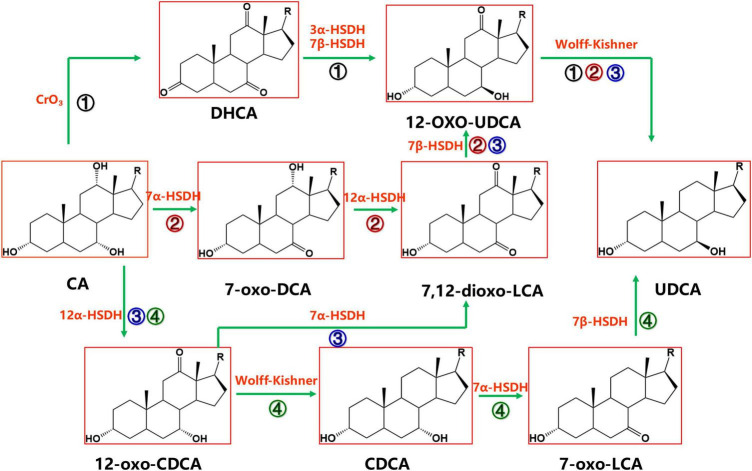
Chemoenzymatic routes for the production of UDCA from CA. DHCA, dehydrocholic acid; 12-oxo-UDCA, 12-oxo-ursodeoxycholic acid; CA, cholic acid; 7-oxo-DCA, 7-oxo-cholic acid; 7,12-dioxo-LCA, 7,12-dioxo-lithocholic acid; UDCA, ursodeoxycholic acid; 12-oxo-CDCA, 12-oxo-chenodeoxycholic acid; CDCA, chenodeoxycholic acid; R, 4-pentynoic acid. ➀, route 1; ➁, route 2; ➂, route 3; ➃, route 4.


Cholic acid is currently the most abundant and inexpensive bile acid. It exists in the bile of cattle, sheep and pigs and is most abundant in bovine bile, with the bile acid content reaching 44 g in 1,000 ml of bovine bile (Scalia et al., 1998). There are four synthetic pathways for the synthesis of UDCA from CA, as shown in Figure 2, but each pathway requires the reduction of the intermediate product C-12 carbonyl to methylene with the help of the Wolff–Kishner reaction (Newman et al., 2014), so the full UDCA biosynthesis process is not yet possible using bile acid as the substrate.

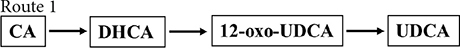


Cholic acid was dehydrogenated (CrO_3_) to produce dehydrocholic acid (DHCA) (Carrea et al., 1992), the C-3 and C-7 carbonyl groups of DHCA were reduced by 3α-HSDH and 7β-HSDH, respectively, to produce 12-oxo-UDCA (Carrea et al., 1992; Bakonyi et al., 2012; Braun et al., 2012; Liu et al., 2012; Sun et al., 2012), and finally 12-oxo-UDCA was synthesized into UDCA by the Wolff–Kishner reaction (Carrea et al., 1992; Bovara et al., 1996; Bakonyi et al., 2012; Braun et al., 2012; Liu et al., 2012; Sun et al., 2012).


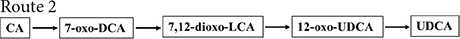


7α-Hydroxysteroid dehydrogenase catalyzed the dehydrogenation of the CA C-7 hydroxyl group to generate 7-oxo-DCA, 12α-HSDH continued to oxidize the C-12 hydroxyl group to a carbonyl group to generate 7,12-dioxo-LCA, 7β-HSDH then reduced the C-7 carbonyl group to a β hydroxyl group to synthesize 12-oxo-UDCA (Bovara et al., 1996; Monti et al., 2009), and 12-oxo-UDCA was finally synthesized into UDCA by the Wolff–Kishner reaction.


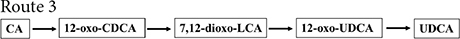


12α-Hydroxysteroid dehydrogenase catalyzed the dehydrogenation of the CA C-12 hydroxyl group to generate 12-oxo-CDCA (Giovannini et al., 2008). 7α-HSDH oxidized the 12-oxo-CDCA C-7 hydroxyl group to a carbonyl group to generate 7,12-dioxo-LCA (Bovara et al., 1996; Monti et al., 2009) and reduced the C-7 carbonyl group to a β hydroxyl group by 7β-HSDH to generate 12-oxo-UDCA (Bovara et al., 1996; Monti et al., 2009). Then, UDCA was finally synthesized by the Wolff–Kishner reaction.


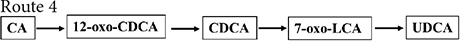


12α-Hydroxysteroid dehydrogenase (Giovannini et al., 2008) catalyzed the dehydrogenation of the CA C-12 hydroxyl group to generate 12-oxo-CDCA, and then the carbonyl group was removed by the Wolff–Kishner reaction to generate CDCA, which entered the CDCA-to-UDCA enzymatic synthesis pathway.

Among the four routes, Route 4 is preferable. In Route 4, UDCA can be synthesized using both CA and CDCA, which are abundant and inexpensive substrates; compared with Route 1, Route 4 cuts one chemical reaction step, offering a greener and more efficient synthesis; compared with Route 2 and Route 3, the 7α-HSDH and 7β-HSDH combination in Route 4 provides a higher substrate conversion rate (Line 127–131).

### LCA as a substrate for UDCA synthesis

**FIGURE 3:**
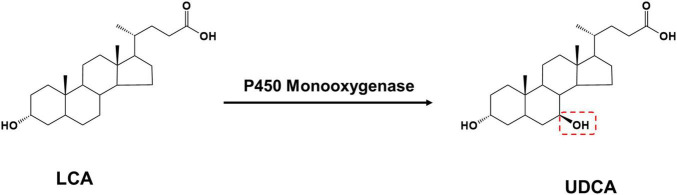
The production of UDCA from LCA catalyzed by P450 monooxygenase.

Lithocholic acid is found in the bile of farm animals such as cattle, sheep, and pigs and is an abundant and inexpensive waste product from meat processing. Based on the structural formula, UDCA synthesis using LCA as a substrate only requires LCA C-7 hydroxylation (Figure 3).

P450 monooxygenases catalyze this reaction; however, P450 enzymes face many challenges in practical applications: their redox catalytic process using O_2_ is highly dependent on the electron-providing cofactor NAD(P)H and the electron transporting reducing chaperone protein, and the complex molecular configuration of this protein reduces P450 enzyme stability and catalytic efficiency. The use of H_2_O_2_ instead of O_2_ eliminates the need for cofactors and their complex electron transport chains, thus simplifying the P450 enzyme catalytic pathway. However, the vast majority of natural P450 enzymes have low or no activity in the presence of H_2_O_2_ (Ma et al., 2018). Currently, only a few microorganisms can convert LCA into UDCA, and the only free enzyme that catalyzes UDCA synthesis from LCA is an engineered cytochrome P450 monooxygenase OleP (Grobe et al., 2020), which was initially found to be involved in the epoxidation reaction in the oleandomycin biosynthetic pathway and was later found to also recognize 12-membered macrolides as substrates. Using protein engineering, the regioselectivity of OleP was further modified to catalyze the conversion of LCA into UDCA: the researchers first docked LCA as a substrate to the OleP protein, analyzed the interaction of the substrate with amino acid residues, and obtained five mutants capable of producing UDCA. Furthermore, a small mutant library containing 4,480 mutants was constructed using the 3DM database (Kuipers et al., 2010), from which a mutant with substantially increased selectivity in the C-7 hydroxylated region was screened for UDCA synthesis.

### Mode of UDCA synthesis catalyzed by free enzymes

Ursodeoxycholic acid synthesis using CDCA as a substrate and catalyzed by free enzymes is currently the most promising biosynthesis method, and CDCA has obvious advantages over the other two substrates in terms of cost, conversion rate and technical maturity. UDCA synthesis using CDCA as a substrate is generally carried out with one-pot, two-step or one-pot, one-step methods. The former method uses a step-by-step reaction, in which 7α-HSDH catalyzes CDCA to produce 7-KLCA in the first step. After that reaction is completed, the enzyme is inactivated (or spatially isolated from the second step), and then 7β-HSDH is added for the second step. If a high temperature was used to inactivate the enzyme after the first step, the temperature needs to be lowered before the second step (Zheng et al., 2015, 2017a). This method has a high conversion rate but a low substrate concentration, low efficiency, and high time consumption. The latter method involves the addition of 7α-HSDH, 7β-HSDH, the substrate and all the auxiliary materials to the reactor together, and the reaction is simultaneously completed in one step. This method has a high substrate concentration, efficiency and energy savings because there is no intermediate step required to raise and lower the temperature, but it is limited by the reaction equilibrium of the enzyme and the low substrate conversion rate, resulting in a low product yield and complicated subsequent purification process (Zheng et al., 2015, 2017a).

A new 7β-HSDH from *Ruminococcus torques* was identified, heterologously expressed in *Escherichia coli* and used together with NADPH-dependent 7α-HSDH from *C. absonum* in a one-pot, one-step reaction (Zheng et al., 2015). The final conversion rate was limited to 73% due to chemical equilibrium, without additional coenzyme regeneration. Later, a one-pot, two-step reaction strategy was used, in which the enzyme involved in the first step was thermally inactivated after the first step (dehydrogenation) to prevent the reduction of 7-KLCA to CDCA in the second step, and the UDCA yield was significantly increased to more than 98% at a substrate loading of 10 mM, and no residual 7-KLCA intermediate was detected.

7α-Hydroxysteroid dehydrogenase from *C. absonum* DSM 599 and 7β-HSDH from *R. torques* ATCC 359157 were used to produce several tens of kilograms of UDCA by the one-pot, one-step method with a conversion rate of more than 90% and a product purity of 98.5%, achieving large-scale UDCA production with CDCA (Zhang et al., 2019).

Almost all of the discovered 7β-HSDHs are NADPH-dependent enzymes, and a wild-type NADH-dependent 7β-HSDH from *L. spicheri* was identified, recombinantly expressed and purified by Tonin et al. (2018). This enzyme, in combination with the NAD^+^-dependent type 7α-HSDH from *Stenotrophomonas maltophilia*, converted CA and CDCA to UCA or UDCA with a >90% yield and only NAD^+^ addition required for catalysis.

To improve the efficiency of HSDHs, enzymes can be immobilized before use, regardless of whether a one-pot, two-step or one-pot, one-step method is used. Yang et al. (2017) used the enzyme immobilization technique to improve the activity and thermal stability of 7α-HSDH and 7β-HSDH, and the catalytic efficiency and reusability of 7α-HSDH and 7β-HSDH coimmobilized at EP-0.5-C were improved: the conversion of the coimmobilized enzymes was increased by approximately 45%, and the conversion remained at 83.7% after seven consecutive cycles.

7α-Hydroxysteroid dehydrogenase/lactate dehydrogenase (LDH) and 7β-HSDH/glucose dehydrogenase (GDH) were immobilized on epoxy resin ES-103 to produce immobilized enzymes LDH-7αHSDH@ES-103 and 7βHSDH-GDH@ES-103, respectively, for catalyzing UDCA synthesis with CDCA (Zheng et al., 2017a). By optimizing the immobilization pH, time, and enzyme–resin loading ratio, the specific activities of LDH-7αHSDH@ES-103 and 7βHSDH-GDH@ES-103 were increased by 12-fold and 516-fold, respectively. Subsequently, continuous UDCA production from CDCA was achieved by using the immobilized enzyme in two continuous packed-bed reactors. In the packed-bed reactor, the UDCA yield was close to 100%, and the reaction duration was at least 12 h, which was superior to that of the batch reaction. The continuous immobilized enzyme method developed in this study may provide a reference for UDCA synthesis by large-scale CDCA bioconversion.

A novel method for *in vitro* tauroursodeoxycholic acid (TUDCA) preparation was taken (Ji et al., 2016): a pair of 7α-HSDH and 7β-HSDH from *C. absonum* DSM599 were coimmobilized using modified chitosan microspheres as a carrier. In the batch reaction catalyzed by the dual-enzyme coupling system, more than 72% of taurochenodeoxycholic acid (TCDCA) was converted. The TUDCA yield achieved by coimmobilized enzyme microsphere catalysis was over 62%, and this method has potential applications in the *in vitro* synthesis of TUDCA and other high-value bile acid derivatives.

### Protein engineering and cofactor regeneration strategies for enzymes used in UDCA synthesis

#### Protein engineering of hydroxysteroid dehydrogenase

To improve the efficiency and conversion rate of enzymatic UDCA synthesis, protein engineering technology has been applied to modify UDCA synthesis enzymes to improve their stability and activity or enhance the tolerance of HSDHs toward high substrate concentrations and even change the coenzyme specificity of HSDHs to reduce the production cost.

A combination of directed evolution and high-throughput screening was used for improving the catalytic efficiency and substrate-concentration tolerance of 7α-HSDH in *C. absonum* (Huang et al., 2019). Compared with the wild type, the best mutant 7α-3 increased the enzymatic activity by 5.5-fold and the catalytic efficiency for CDCA and NADP^+^ by 10-fold and 14-fold, respectively. In addition, the tolerance of 7α-3 to high substrate concentrations was significantly enhanced compared with that of the wild type. Due to the improved catalytic efficiency and enhanced substrate-concentration tolerance, 7α-3 converted 100 mM CDCA to 7-KLCA with a 99% conversion rate in 2 h. The conversion rate of the wild type under the same conditions was only 85% in 16 h.

Error-prone PCR, DNA shuffling, and site mutation were used to target and modify 7β-HSDH from *R. torques*, improve the activity, thermal stability, and optimal pH of 7β-HSDH (Zheng et al., 2017b). The best mutant, V3-1, had a 5.5-fold higher specific activity and a 28-fold longer half-life than the wild type. In addition, the optimal reaction pH of the mutant changed from acidic to weakly basic. In the cascade reaction, the yield of UDCA synthesized by the V3-1 mutant increased to 942 g L^–1^ d^–1^ compared with 141 g L^–1^ d^–1^ for the wild type. This study provides a useful strategy for the modification of HSDHs to promote UDCA biosynthesis.

Almost all existing reported 7β-HSDH are strictly dependent on NADPH; however, NADH is more economical than NADPH and is a preferred cofactor for HSDH synthesis in UDCA applications. You et al. (2018) used a strategy called “Cofactor specificity reversal: small-and-smart library design (CSR-SaSLiD) strategy” to coengineer recombinant 7β-HSDH derived from *R. torques* to alter its cofactor dependence. They designed a CSR-SaSLiD library consisting of only five mutants, resulting in the successful discovery of two ideal 7β-HSDH mutants, G39D and G39D/T17A. G39D showed a 953,000-fold reversal in NADH/NADPH cofactor specificity, while G39D/T17A showed a 223-fold increase in activity against NADH. Through molecular dynamics simulations, they elucidated the structural mechanism by which mutations reverse cofactor preference and increase catalytic activity and further demonstrated that the CSR-SaSLiD strategy can be extended to other 7β-HSDHs. This work provides an efficient method for altering the cofactor preference of HSDHs and subsequently restoring enzyme activity and can reduce the cost of transformation.

An NADP^+^-dependent 7α-HSDH gene from *Clostridium difficile* was cloned and expressed in *E. coli* (Bakonyi and Hummel, 2016). It was found that unlike other 7α-HSDHs, this enzyme did not exhibit substrate inhibition. By site mutation based on structural analysis, they transformed the cofactor specificity of this enzyme to accept NAD(H), thereby reducing the cost of using cofactors in UDCA synthesis.

Lou et al. (2017) identified the cofactor-specific determining sites (csss) of 7α-HSDH from *C. absonum*, namely T15, R16, R38, and R194, and then performed alanine scanning mutagenesis and molecular dynamics (MD) simulations. The results showed that the csss of wild-type 7α-HSDH had high affinity for NADP(H) but low catalytic efficiency (*k*_cat_) for NADP^+^. However, the mutant R194A had a higher catalytic efficiency (*k*_cat_/K_m_) for NADP^+^ than the wild type and other mutants, which increased the catalytic efficiency of R194A toward CDCA oxidation to more than three times that of the wild type.

In addition, several patents have reported numerous evolutionary studies and related applications of HSDH enzymes, also focusing on enzyme activity enhancement, stability enhancement, high-substrate-concentration tolerance improvement or cofactor-specific transformation (Weuster-botz et al., 2017, 2021; Liu et al., 2018).

#### Cofactor regeneration strategy

The enzymes that catalyze the biosynthesis of UDCA from CDCA or CA are HSDHs, and they all belong to the family. One of the typical features of this family of enzymes is that they have an NAD(P)(H) binding region (TGXXX[AG]XG), and their reactions all require NAD(H) or NADP(H) coenzymes (Figure 1; Kavanagh et al., 2008; Savino et al., 2016; Wang et al., 2017). Theoretically, equimolar amounts of cofactors are required in relation to the amount of substrate that is consumed or product that is generated, and these cofactors are often expensive, making it infeasible to increase the cofactor amount from an economic point of view; on the other hand, excess cofactors also inhibit HSDH activity, which technically limits cofactor application. Therefore, according to the process economics of biocatalytic reactions and the feasibility of industrial applications, HSDH systems must meet the requirements of efficient and low-cost cofactor regeneration systems in addition to having suitable enzymes and compatible reaction engineering technologies in their applications. Cofactor regeneration refers to the regeneration of the cofactor from the oxidation state to the reduction state or vice versa, which allows the cofactor to be maintained at a certain catalyst amount level. A number of methods have been proposed to address this cofactor regeneration issue, including chemical, photochemical, enzymatic, and electrochemical regeneration systems (Zhang, 2001; Hummel and Gröger, 2014). Enzymatic UDCA synthesis must also take advantage of a suitable cofactor regeneration system for industrial UDCA production. The efficiency of a cofactor regeneration system is usually measured by the cofactor conversion number (turnover number, TN) and the total conversion number (total turnover number, TTN) (Weckbecker et al., 2010; Kara et al., 2014; Mordhorst and Andexer, 2020). Bioconversion processes with TTNs of 10^3^–10^5^ are usually considered economically feasible. Coenzyme I (NAD^+^) and coenzyme II (NADPH) in enzymatic UDCA synthesis are almost always regenerated enzymatically, and dehydrogenases are used (Table 3).

**TABLE 3:** The dehydrogenases used for cofactor regeneration in biological synthesis of UDCA.

Dehydrogenase	NAD^+^ or NADPH regeneration	Application	References
Lactate dehydrogenase (LDH) and glucose dehydrogenase (GDH)	LDH for NAD^+^, GDH for NADPH	Conversion of CDCA into UDCA	Zheng et al., 2015
Alcohol dehydrogenase (ADH)	NADPH	Conversion of 7-KLCA into UDCA	Liu et al., 2011
Formate dehydrogenase (FDH)	NADH	Synthesis of ursolic acid (UCA) and UDCA	Pedrini et al., 2005
Glucose dehydrogenase (GDH)	NADPH	Conversion of 7-KLCA into UDCA	Zheng et al., 2017b
Malate dehydrogenase (MDH)	NADH	Conversion of 7-KLCA into UDCA	Qin et al., 2018
Flavin oxidoreductase (FR) and alcohol dehydrogenase (ADH)	FR for NAD^+^, ADH for NADPH	Conversion of CDCA into UDCA	Chen et al., 2019
Glutamate dehydrogenase (GLDH) and glucose dehydrogenase (GDH)	GLDH for NADP^+^, GDH for NADPH	Conversion of CA into 12-keto-UDCA	Bovara et al., 1996
D-type amino acid dehydrogenase (DAADH)	NADP^+^	Conversion of CDCA into 7-KLCA	Huang et al., 2019
Glucose dehydrogenase (GDH)	NADH and NADPH	Conversion of dehydrocholic acid (DHCA) into 12-keto-ursodeoxycholic acid (12-keto-UDCA)	Sun et al., 2012
Glutamate dehydrogenase (GLDH)	NADP^+^	Conversion of CA into 12-keto-CDCA	Carrea et al., 1985

As listed in Table 3, there are a variety of dehydrogenases available for NAD(H) and NADP(H) regeneration in the biological synthesis of UDCA. However, some commonly used dehydrogenases have notable characteristics (Table 4): the most commonly used are LDH for NAD^+^ regeneration and GDH for NADPH regeneration. One of the reasons for this is that coenzyme NAD^+^ regeneration using the LDH/pyruvate system is more advantageous in terms of cost compared to other coenzyme regeneration systems; LDH is more active and more stable in the reaction system, and the reaction substrate pyruvate is relatively inexpensive. In conclusion, the LDH/pyruvate system is advantageous for NAD^+^ regeneration because of its low cost, high stability and easy post-processing. Regarding NADPH regeneration, the GDH/glucose system has very outstanding features, including being extremely stable to oxygen and having a highly specific activity for NADP^+^. Moreover, glucose is inexpensive, easy to obtain, stable, highly reducible, harmless to enzymes and coenzymes, and increases enzyme stability. These advantages make GDH/glucose the best dehydrogenase system for NADPH regeneration. Although the byproduct gluconate produced by the GDH/glucose coenzyme regeneration system may complicate the post-reaction treatment, this system still has significant advantages over other coenzyme regeneration systems in terms of cost and stability in large-scale applications. The GDH/glucose regeneration system is currently the best method for regenerating NADPH.

**TABLE 4:** Commonly used cofactors regeneration system.

Dehydrogenase	Coenzyme regeneration mode	Reaction	Advantages and disadvantages	References
Lactate dehydrogenase (LDH)	NAD^+^ regeneration	Reduction of pyruvate to L-lactate	High enzyme specific activity; low enzyme cost; low redox capacity; most cannot regenerate NADP^+^	Nathalie et al., 1991; Zhou and Shao, 2010
Glutamate dehydrogenase (GLDH)	NAD(P)^+^ regeneration	Reduction of α-ketoglutarate to L-glutamate	Commonly used coenzyme regeneration system; high enzyme cost	Carrea et al., 1985; Tian et al., 2017
Glucose dehydrogenase (GDH)	NAD(P)H regeneration	Oxidation of glucose to gluconic acid	Good stability; high specific activity for both NAD^+^ and NADP^+^; viscous product gluconic acid increasing the difficulty of product separation	Nagao et al., 1992; Xu et al., 2006
Formate dehydrogenase (FDH)	NADH regeneration	Oxidation of formate to carbon dioxide	The product carbon dioxide is non-toxic to enzymes and easy to remove from reaction system; the reaction is irreversible; low enzyme activity and high enzyme cost; poor specificity and sensitivity to organic solvents	Allen and Holbrook, 1995
Alcohol dehydrogenase (ADH)	NAD(P)H regeneration	Oxidation of alcohols to aldehydes (ketones)	Alcohols and aldehydes (ketones) are volatile and easily removed from the reaction system; low redox capacity; if alcohols and aldehydes (ketones) are not removed in time, enzyme activity will be inhibited	Hollmann et al., 2005; Höllrigl et al., 2008; Pennacchio et al., 2008

## Whole-cell UDCA synthesis

Research on whole-cell UDCA synthesis began with the discovery that wild microbial strains convert CDCA or LCA to UDCA, and researchers found that some microorganisms convert CDCA to UDCA when cultured alone (Macdonald et al., 1981; Kole and Altosaar, 1985; Lepercq, 2004) or in coculture (Hirano and Masuda, 1981; MacDonald et al., 1982); however, wild microorganisms can only carry very low substrate concentrations (micromolar or a few millimolar CDCA), and once the substrate concentration increases, microbial growth is inhibited. Moreover, their conversion rate is also very low, with gram-level substrate concentrations being converted into only milligram-level product concentrations. It was later confirmed that these microorganisms self-express 7α-HSDH and/or 7β-HSDH.

For the whole-cell catalytic study of converting LCA into UDCA, Sawada et al. (1982) isolated 609 fungal strains with hydroxylation abilities from soil samples, from which a strain of *Fusarium* spp. *Fusarium equiseti* M41 with good C-7 hydroxylation ability was selected, and this strain converted 1 g/L LCA into 350 mg/L UDCA after 112 h of incubation. Previously, Carlström et al. (1981) also reported that *Absidia coerulea* and *Rhizoctonia solani* also had the ability to hydroxylate C-7.

Kollerov et al. (2013) further screened different species of actinomycetes and filamentous fungi for their ability to convert LCA into UDCA and identified for the first time that *Bipolaris, Gibberella, Cunninghamella, Curvularia, Pseudonocardia, Saccharothrix, Amycolatopsis, Lentzea, Saccharopolyspora*, and *Nocardia genera* directly converted LCA into UDCA. Moreover, they screened a *Gibberella zeae* VKM F-2600 strain with high β-hydroxylation activity at C-7 against LCA. After optimizing the conditions, close to 90% of 1 g/L LCA was converted into UDCA. Recently, a mutant *G. zeae* M23 was obtained by UV mutagenesis (Kollerov and Donova, 2022). The mutant strain offered better conversion ability and could convert 88% of 4 g/L LCA into UDCA. This yield of UDCA is superior to previously reported yields.

The microorganisms that convert CDCA to UDCA produce 7α-HSDH and 7β-HSDH, and the microorganisms that convert LCA to UDCA produce P450 monooxygenases. These microorganisms became an important source of late HSDH genes and protein sequences. However, wild microorganisms, because of their low HSDH expression, have low enzyme activities, and slightly higher concentrations of substrate also inhibit their growth and conversion. Therefore, relevant whole-cell catalytic studies were little explored by researchers. It was not until the heterologous expression of related HSDHs using engineered strains, especially *E. coli*, and the more successful synthesis of UDCA from CDCA with these engineered bacteria that whole-cell catalysis research came back into the limelight. Compared with free-enzyme catalysis, whole-cell catalysis is advantageous because it uses the coenzyme regeneration systems of microbial cells and eliminates the steps of bacterial crushing and enzyme extraction. Regarding the synthesis of UDCA from LCA, progress has not yet been made in whole-cell catalysis research because of the lack of P450 enzyme resources and harsh reaction conditions.

An engineered *E. coli* integrating 7α-HSDH and 7β-HSDH had been constructed (Shi et al., 2017) to convert TCDCA into TUDCA and CDCA into UDCA. The recombinant *E. coli* integrating *Clostridium sardiniense*-derived 7α-HSDH and *Ruminococcus gnavus*-derived 7β-HSDH was able to convert approximately 50% of TCDCA to TUDCA, and this study provided a potential pathway for producing a bear bile substitute using inexpensive and readily available chicken bile. Later, they developed a deep tank static process through fermentation optimization and balanced bidirectional reactions to control the ratio of converted TUDCA to TCDCA similar to that of medicinal bear bile powder, providing a practical yet environmentally friendly industrial production method for producing artificial bear bile powder substitutes from chicken bile powder using microbial cell factories (Xu et al., 2019). Recently, the group constructed a *Saccharomyces cerevisiae* host expressing these two enzymes and optimized the gene combination and whole-cell transformation conditions. From the perspective of food and drug safety, *S. cerevisiae* is a GRAS strain and has a higher safety profile than *E. coli* (Jin et al., 2022).

The whole-cell synthesis of 12-keto-UDCA, a key intermediate in the chemical-enzymatic synthesis of UDCA, had been achieved (Sun et al., 2012). 7β-HSDH mutant from *Collinsella aerofaciens* and 3α-HSDH from *Comamonas testosteroni* were used to reduce DHCA to 12-keto-UDCA by a two-step reaction. Researchers first coexpressed 3α-HSDH, 7β-HSDH, and a formate dehydrogenase (FDH) mutant from *Mycobacterium vaccae* N10 in the same strain of *E. coli* BL21(DE3) cells, thus constructing a single-strain whole-cell catalytic system; then, they coexpressed 3α-HSDH, 7β-HSDH and a GDH from *Bacillus subtilis* in the same strain of *E. coli* BL21(DE3) cells to construct another single-strain whole-cell catalytic system. After comparing the two whole-cell catalytic systems, the single-strain expression system constructed by 3α-HSDH, β-HSDH, and GDH achieved the best results, and the system was able to almost completely convert 100 mM DHCA into 12-keto-UDCA (>99.5 mM) within 4.5 h.

Braun et al. (2012) similarly achieved the whole-cell synthesis of 12-keto-UDCA. They expressed NADH-dependent 3α-HSDH from *C. testosteroni* in an *E. coli* strain already overexpressing 7β-HSDH (from *C. aerofaciens*) and an NAD(P) bispecific FDH mutant (from *M. vaccae*). This engineered cell was able to directly convert 50 mM DHCA to 12-keto-UDCA with only a small amount of intermediate product remaining.

The complete conversion of 30 mM CDCA into UDCA in one pot had been achieved, using different coenzyme-specific 7α-HSDH and 7β-HSDH and an independent coenzyme cycling system (Chen et al., 2019). In the study, an NAD^+^ regeneration system was developed by using flavin oxidoreductase (FR) and flavin mononucleotide (FMN). The FR/FMN system subsequently coupled NAD^+^-dependent 7α-HSDH from *Brevundimonas* sp. to oxidize CDCA to 7-KLCA. When whole cells of *E. coli* coexpressed 7α-HSDH and FR, a complete conversion of 50 mM CDCA into 7-keto-LCA was achieved. For the reduction of 7-KLCA, NADPH-dependent 7β-HSDH from *Clostridium* sp. was used, and alcohol dehydrogenase from *Thermoanaerobacter brockii* (TbADH) and isopropanol were used as cofactor regeneration systems. When catalyzed by this *E. coli*-engineered bacterial whole-cell system, 40 mM 7-KLCA was reduced to UDCA with a conversion rate of 26.8%. Then, 12.5 mM CDCA was completely converted into UDCA when both cells were reacted simultaneously in a one-pot, one-step method. Unfortunately, bile acids, especially UDCA, inhibit the activity of these enzymes, so the high concentration of substrate and intermediate products prevented the complete conversion of 7α-OH, and the substrate inhibition problem still needs to be solved by enzyme engineering.

Regarding whole-cell catalysis, several patented technologies have been granted. Weuster-botz et al. (2017) reported the recombinant whole-cell biocatalytic production of UDCA with a 7β-HSDH enzyme mutant in patent US 20170191104; Hummel and Bakonyi (2022), in turn, provided a novel 3α-HSDH mutant and a method for UDCA production using a recombinant microorganism containing this mutant.

The biological synthesis modes of UDCA are summarized in Table 5. In short, each of these synthesis methods has different characteristics. In terms of synthesis efficiency, CDCA is the most efficient substrate and has the highest conversion rate in both enzymatic and whole-cell synthesis methods, which is the focus of current research.

**TABLE 5:** An overall comparison of biological synthesis methods for UDCA production.

Synthesis mode	Enzyme or cell	Substrates	Substrate load (mM)	Conversion yield (%)	Features
Free enzyme-catalyzed synthesis	7α-HSDH and 7β-HSDH	CDCA	>100	>90	Achieved conversion only depends only on enzymes; enzymes have high catalytic efficiency; is the only biosynthesis mode to achieve industrial production of UDCA
	3α-HSDH, 7α-HSDH, 12α-HSDH, 7β-HSDH	CA	>100	<70	Intermediate synthesis requires chemical reaction steps; the cost of chemical reaction is high; conversion yield is low
Whole-cell synthesis	Mainly engineered *E. coli* or yeast	CDCA	<50	>90	Could reduce the consumption of coenzymes and decrease costs
	Mainly wild fungi or their mutants	LCA	<10	<90	The concentration of loading substrate is low; a high concentration of substrate inhibits the cell growth and conversion rate

## Conclusion and future perspectives

With the continuous application development of UDCA and its derivatives in the fields of hepatobiliary diseases and tumor therapy in recent years, UDCA synthesis research has made great strides. Currently, chemical synthesis is still the main method for large-scale UDCA production, but this method is not sufficient for meeting the market demand due to factors such as low yield, cumbersome steps and environmental unfriendliness. Biosynthesis, on the other hand, has gradually become the mainstream research direction for UDCA preparation because of its advantages of a high conversion rate, low byproduct levels and environmental safety.

Ursodeoxycholic acid biosynthesis has demonstrated its superiority over chemical UDCA synthesis, and the industrial UDCA production process using CDCA as substrate has been realized. However, there are still some limitations of this process in actual production. First, the enzymes used (especially 7β-HSDH) are unstable, and their enzyme activities are rapidly reduced in the reaction system. Second, the substrate-concentration tolerance of enzyme reaction systems is still low, and some enzymes are seriously inhibited by the products or intermediates. A one-pot, one-step method can allow the substrate concentration to be increased, but the conversion rate is low, and the residual substrate and intermediate products put more pressure on the downstream purification; a one-pot, two-step method can increase the conversion rate, but it also requires the substrate concentration to be lowered, resulting in a low production efficiency. It also generates a large amount of wastewater, which results in the risk of environmental harm. Although the expression of existing HSDHs by *E. coli* heterologous expression systems is considerable and the enzyme source is no longer a major limiting factor, mechanical shear stress, and physical damage during the production process can easily cause enzyme loss and lead to a decrease in enzyme recovery. In addition, the potential of coenzyme dosage optimization and whole-cell catalysis in industrial applications still needs to be further explored; the biotransformation of LCA to UDCA is still far from being industrially relevant because of the actual catalytic capacity limitation of the P450 enzyme monooxygenase.

The abovementioned problems are the main factors limiting industrial UDCA production by various biotransformation methods. In addition, the biosynthetic pathway sometimes involves the issue of microbial pathogenicity assessment, which requires the development of efficient heterologous expression systems other than *E. coli* systems. There is no doubt that enzymes are the most central component of the biosynthetic UDCA process, so the further expansion of enzyme sources, the development of screening procedures for new enzymes, and the enhancement of efforts to modify enzymes in the field of protein engineering should result in biocatalysts that can be applied at high substrate concentrations of >100 mM. In contrast, the application of immobilized enzyme technology and the use of recombinant whole-cell catalysis should greatly reduce the cost of catalysis and facilitate product isolation and purification.

The problems to be solved in order to achieve UDCA biosynthesis are as follows: 1. developing one-pot, one-step and whole-cell catalytic methods by mining or modifying cofactor-specific HSDH and cofactor regeneration enzymes while also taking into account the enzyme activity and substrate high-carrying-capacity requirements; and 2. screening wild strains from bacteria and actinomycetes that can transform LCA into UDCA, selecting the P450 monooxygenases with C-7 hydroxylation functions, and further modifying them by protein engineering technology to obtain enzymes that can meet industrial application requirements and make full use of LCA raw material resources.

## Author contributions

PS and RF: conceptualization. XZ, WF, WX, CW, SX, and SY: literature search. PS and XZ: writing—original draft preparation. PS, WX, and SY: writing—review and editing. SX and RF: funding acquisition. All authors read and approved the manuscript.
